# Differential requirement of bone morphogenetic protein receptors Ia (ALK3) and Ib (ALK6) in early embryonic patterning and neural crest development

**DOI:** 10.1186/s12861-016-0101-5

**Published:** 2016-01-19

**Authors:** Carolin Schille, Jens Heller, Alexandra Schambony

**Affiliations:** Biology Department, Developmental Biology, Friedrich-Alexander University Erlangen-Nuremberg, 91058 Erlangen, Germany

**Keywords:** BMP receptor, ALK3, ALK6, *Xenopus*, Dorso-ventral patterning, Neural crest

## Abstract

**Background:**

Bone morphogenetic proteins regulate multiple processes in embryonic development, including early dorso-ventral patterning and neural crest development. BMPs activate heteromeric receptor complexes consisting of type I and type II receptor-serine/threonine kinases. BMP receptors Ia and Ib, also known as ALK3 and ALK6 respectively, are the most common type I receptors that likely mediate most BMP signaling events. Since early expression patterns and functions in *Xenopus laevis* development have not been described, we have addressed these questions in the present study.

**Results:**

Here we have analyzed the temporal and spatial expression patterns of ALK3 and ALK6; we have also carried out loss-of-function studies to define the function of these receptors in early *Xenopus* development. We detected both redundant and non-redundant roles of ALK3 and ALK6 in dorso-ventral patterning. From late gastrula stages onwards, their expression patterns diverged, which correlated with a specific, non-redundant requirement of ALK6 in post-gastrula neural crest cells. ALK6 was essential for induction of neural crest cell fate and further development of the neural crest and its derivatives.

**Conclusions:**

ALK3 and ALK6 both contribute to the gene regulatory network that regulates dorso-ventral patterning; they play partially overlapping and partially non-redundant roles in this process. ALK3 and ALK6 are independently required for the spatially restricted activation of BMP signaling and *msx2* upregulation at the neural plate border, whereas in post-gastrula development ALK6 exerts a highly specific, conserved function in neural crest development.

**Electronic supplementary material:**

The online version of this article (doi:10.1186/s12861-016-0101-5) contains supplementary material, which is available to authorized users.

## Background

Bone morphogenetic proteins (BMPs) are a subfamily of the TGFβ superfamily of secreted growth factors. Originally identified as regulators of bone formation [1–3] it is now well established that BMPs are major factors of dorso-ventral axis determination from *Drosophila* to mammals [4–10]. During later development, BMPs contribute to neural patterning and differentiation, the induction of the vertebrate neural crest and placodes and the development of the lens and inner ear [11–18]. BMP signaling is also required in eye development, cardiac, kidney, and thymus organogenesis, germ cell differentiation, and hematopoiesis [19–25]. In addition, its roles in chondrogenesis, skeletal and limb development and patterning are well documented [reviewed in 26–29].

BMP ligands bind to and activate a tetrameric receptor complex composed of two type I and two type II receptors [30–34]. Type I receptors are also referred to as Activin-receptor Like Kinase (ALK). The human genome contains 35 Transforming Growth Factor beta (TGFβ) family genes, but only seven genes encoding for type I, and five genes encoding for type II receptors. BMP receptors are single-pass transmembrane proteins and possess a serine/threonine kinase domain in their intracellular domains [35–40]. BMP ligands activate only a subset of these receptors, namely BMP Receptor II (BMPRII) or Activin Receptor IIB (ActRIIB) as type II receptors and BMP Receptor Ia (BMPRIa/ALK3), BMP Receptor Ib (BMPRIb/ALK6) or Activin Receptor Ia (ActRIa/ALK2) as type I receptors; in some cases, the Activin Receptor Ib (ActRIb/ALK4) is also activated [41, 42]. Upon ligand binding the type II receptors phosphorylate and activate the type I receptors. In the canonical TGFβ signal transduction pathway, activated type I receptors phosphorylate a receptor substrate protein of the Mad/Smad family of transcriptional regulators (R-Smads). Phosphorylated Smads bind another Smad family member, the so-called Co-Smad, which itself is not a substrate of the type I receptor; they are then imported into the nucleus. Nuclear Smads associate with additional transcriptional regulators and cofactors, and regulate transcription of their target genes [reviewed in 43]. R-Smads are subdivided into two groups, the Smad 2/3 and the Smad 1/5/8 groups, which mediate signaling by TGFβ, Activin and Nodal ligands or by BMPs, respectively [reviewed in 44].

In *Xenopus laevis*, BMP4 is expressed in a semicircle at the ventral side of the embryo at early gastrula stage 10.5 and plays a major role in the induction of ventral cell fates [7, 45]. Ventral BMP activity is antagonized by secreted BMP antagonists such as chordin or noggin that are derived from the organizer, thereby creating a BMP gradient that patterns the dorso-ventral axis [reviewed in 46]. Similar to ventral BMP4 expression in *Xenopus*, graded expression of BMP ligands is involved in dorso-ventral patterning in zebrafish [47]. BMP7, BMP2 and Anti Dorsalizing Morphogenetic Protein (ADMP) also contribute to ventral cell fate specification in *Xenopus* embryos, although these BMP ligands are expressed without dorso-ventral bias or even exclusively dorsal in the Spemann’s organizer [6, 48–51]. In post-gastrula embryos, BMP ligands are expressed in specific, spatially restricted patterns that reflect their role in the development of the respective tissues and organs. [6, 45, 48]. Similarly, in tadpole embryos expression of BMPR1a is found in the eye, otic vesicle, kidney, branchial arches, foregut and intersomitic tissue. BMPR1b expression is also detected in the head but not in trunk tissue [52–54].

By contrast, little is known about the expression patterns of BMP receptors in early *Xenopus* embryos. Here, we have analyzed the expression pattern and function of BMPRIa/ALK3 and BMPRIb/ALK6 in early *Xenopus* development. We observed only partial functional redundancy of the receptors in dorso-ventral patterning. In late-gastrula and neurula stages the expression patterns and function diverge markedly. In particular, we identified a specific, non-redundant function of ALK6 in neural crest development.

## Results and discussion

### Expression of *bmprIa/alk3* and *bmprIb/alk6* during *Xenopus* development

**Fig. 1:**
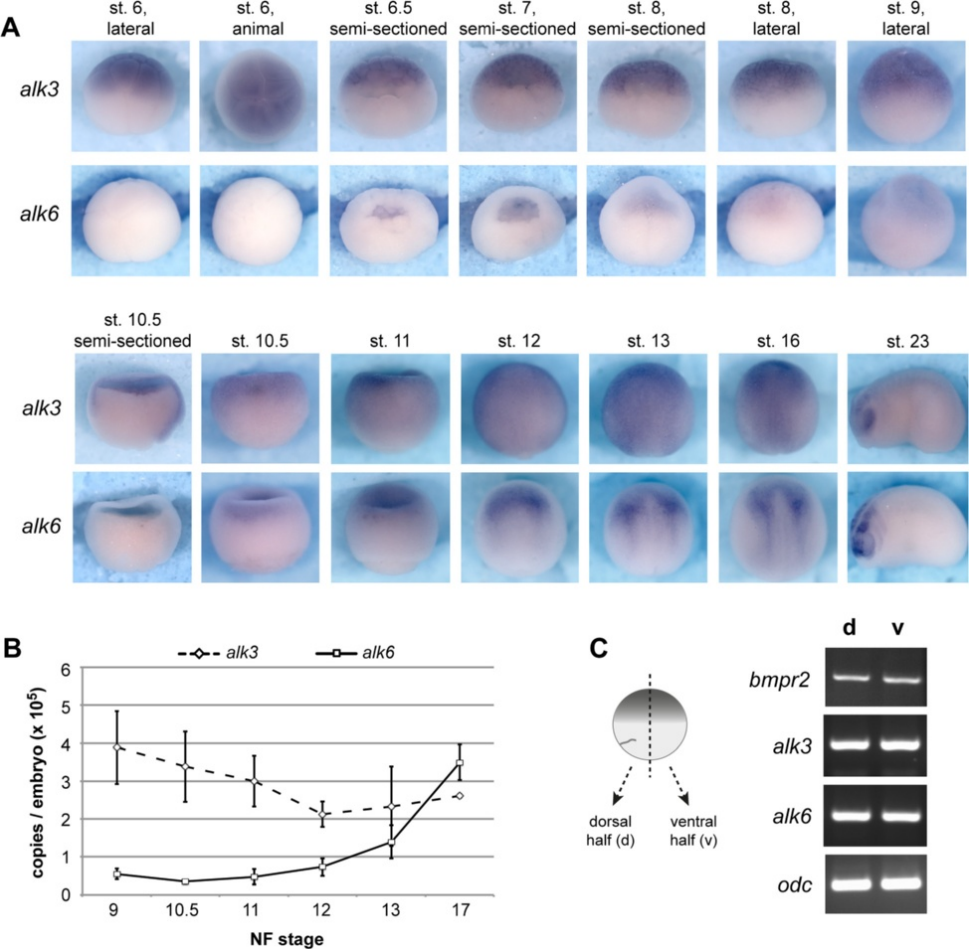
Expression patterns of *alk3* and *alk6* in early *Xenopus laevis* embryos. **a** Temporal and spatial expression patterns were determined by whole-mount in situ hybridization at the indicated Nieuwkoop and Faber (NF) stages. **b** Expression of *alk3* and *alk6* was quantified by Real-Time RT-PCR at the indicated stages; corresponding synthetic sequences were used a external standards for copy number determination. The graph shows the copy number per embryo. **c** NF stage 10.5 embryos were bisected into dorsal and ventral halves and transcripts of the indicated genes were detected by RT-PCR

We have cloned the full-length coding sequence of *Xenopus laevis* BMP-receptor Ia (BMPRIa/ALK3) and BMP-receptor Ib (BMPRIb/ALK6; see Additional file 1: Figure S1 for alignment of the ALK6 sequence and phylogenetic analysis, phylogeny data is also available at http://purl.org/phylo/treebase/phylows/study/TB2:S18663). First, we compared their temporal and spatial expression pattern in early *Xenopus* embryos. In whole-mount in situ hybridization, *alk3* RNA was detected in the animal hemisphere of early to late-blastula and early gastrula stage embryos. Its distribution expanded vegetally, but remained excluded from the vegetal pole, in mid-gastrula stage embryos (Fig. 1a). Notably, no dorso-ventral bias was observed. ALK6 RNA was not detected in blastula stages and only very weakly in early and mid-gastrula stage embryos. By late-gastrula, at Nieuwkoop and Faber stage 12 (NF stage 12, [55]) the spatial expression patterns of *alk3* and *alk6* became divergent. While *alk3* expression remained ubiquitious, *alk6* was specifically upregulated in the anterior neural plate. This temporal and spatial expression pattern is conserved between *Xenopus* and zebrafish gastrulae [56]. In the mouse, *alk3* is also ubiquitously expressed until E9.5, whereas *alk6* was detected only from E9.5 onwards and showed a more restricted expression pattern when compared to *alk3* [57].

In neurula stages, *alk3* was still ubiquitously expressed, but we observed a slightly more intense staining for *alk3* at the neural plate border in NF stage 13 embryos and in the anterior neural plate in NF stage 16 embryos. Again, *alk6* showed a strikingly different expression pattern during neurulation with the strongest expression at the neural plate border, in the anterior neural plate and in the prospective neural crest (Fig. 1a). The neural crest is induced at the neural plate border by a combination of intermediate levels of BMP signaling, WNT and FGF signaling [reviewed in 14, 58, 59]. Among the gene network that defines the neural crest, a subset of genes has been identified as direct targets of the BMP pathway via Smads, including the neural plate border specifying gene *msx2* [60] and *slug* [61]. Therefore the restricted expression pattern of ALK6 in the prospective neural crest suggests a specific role of *alk6* in neural crest specification and development.

In early tadpole stages, expression of *alk3* was detected in the brain and the eye, corresponding to the upregulation in the anterior neural plate at NF stage 16. By contrast, at NF stage 23, *alk6* was strongly expressed in the brain, the ventral part of the eye and in the migrating neural crest cells (Fig. 1a). In later tadpole stages we observed expression of both genes in the brain, the eye, the otic vesicle and the branchial arches, *alk3* transcripts were additionally detected in the heart, in intersomitic tissue and the pronephros (Additional file 2: Figure S2). *Alk6* was detected in the brain, eye, otic vesicle and branchial arches, although in a more spatially restricted pattern than *alk3*. In contrast to an earlier report [52], we also detected *alk6* expression in the notochord (Additional file 2: Figure S2). These late expression patterns mostly recapitulate tadpole expression of both type I BMP receptors, as reported in other studies [52–54]. In addition, the tadpole expression showed considerable overlap with the expression of BMPRII [52, 62].

The increased expression of *alk6* from late-gastrula onwards, which was detected by in situ hybridization (Fig. 1a), correlated well with the expression levels of *alk3* and *alk6* determined by Real-Time RT-PCR (Fig. 1b). Expression levels of *alk3* decreased by 50 % from NF stage 9 to NF stage 12 and then remained constant until stage 17. By contrast, *alk6* levels were 6 to 10-fold lower than *alk3* from NF stage 9 to 11 and showed a marked increase from NF stage 12 to 17, which resulted in an overall higher expression of *alk6* when compared to *alk3* at NF stage 17 (Fig. 1b). In addition, RT-PCR analysis of semi-sectioned NF stage 10.5 embryos confirmed that *alk3*, *alk6* and also the type II receptor *bmpr2* were expressed equally in the dorsal and ventral halves (Fig. 1c). The same was true for the activin receptors *acvr1a/alk2*, *acvr1b/alk4* and *acvr2* at that stage (see Additional file 3: Figure S3).

The expression pattern of both *alk3* and *alk6* in pre- and early gastrula stage embryos mirrors the unbiased expression of BMPRII, BMP2 and BMP7 [6, 48, 62]. According to current knowledge, BMP2 and BMP7 and the ventrally expressed BMP4 activate ventralizing BMP signaling in early embryos. The dorso-ventral gradient of BMP activity found in late blastula and early gastrula *Xenopus* embryos [63] is shaped by ventrally expressed positive regulators and BMP antagonists arising from the dorsal organizer [reviewed in 46]. ALK3 and ALK6 can act as receptors for BMP2, BMP4 and BMP7 [31, 32, 34, 64]; therefore, they are likely to mediate signaling from all three ligands in early embryos.

To further elucidate the role of ALK3 and ALK6 we have performed loss-of-function studies, focusing on pre-gastrula development and characterizing a putative specific role of ALK6 in the neural crest.

### Knock-down of ALK3 and ALK6 results in partially dorsalized embryos and additional specific phenotypes

To investigate the role of ALK3 and ALK6 in *Xenopus* development, we have designed two translation-blocking antisense Morpholino oligonucleotides (MOs), which target non-overlapping sequences to knock-down each protein (see also Additional file 4: Figure S4).

We injected ALK3 MO or ALK6 MO into both blastomeres of two-cell stage embryos and analyzed the resulting phenotypes at NF stage 40. First, we noticed that knock-down of ALK6, particularly with ALK6 MO1, caused a higher lethality of embryos when compared to injection of ALK3 MO or control MO. At neurula stages, 63 of 147 embryos out of three independent injections with ALK6 MO1 survived whereas the numbers of surviving embryos injected with ALK6 MO2 (98/134), ALK3 MO1 (50/68) or ALK3 MO2 (97/122) were similar to the control MO (92/137). At stage 40, the survival of ALK6 MO1 and ALK6 MO2 injected embryos dropped to 17 ± 13 (total 28/147 embryos) and 26 ± 13 % (total 42/134 embryos) respectively. By comparison, 50–56 % embryos survived to stage 40 after injection of either control MO, ALK3 MO1 or ALK3 MO2 (see also Additional file 5: Figure S5).

**Fig. 2:**
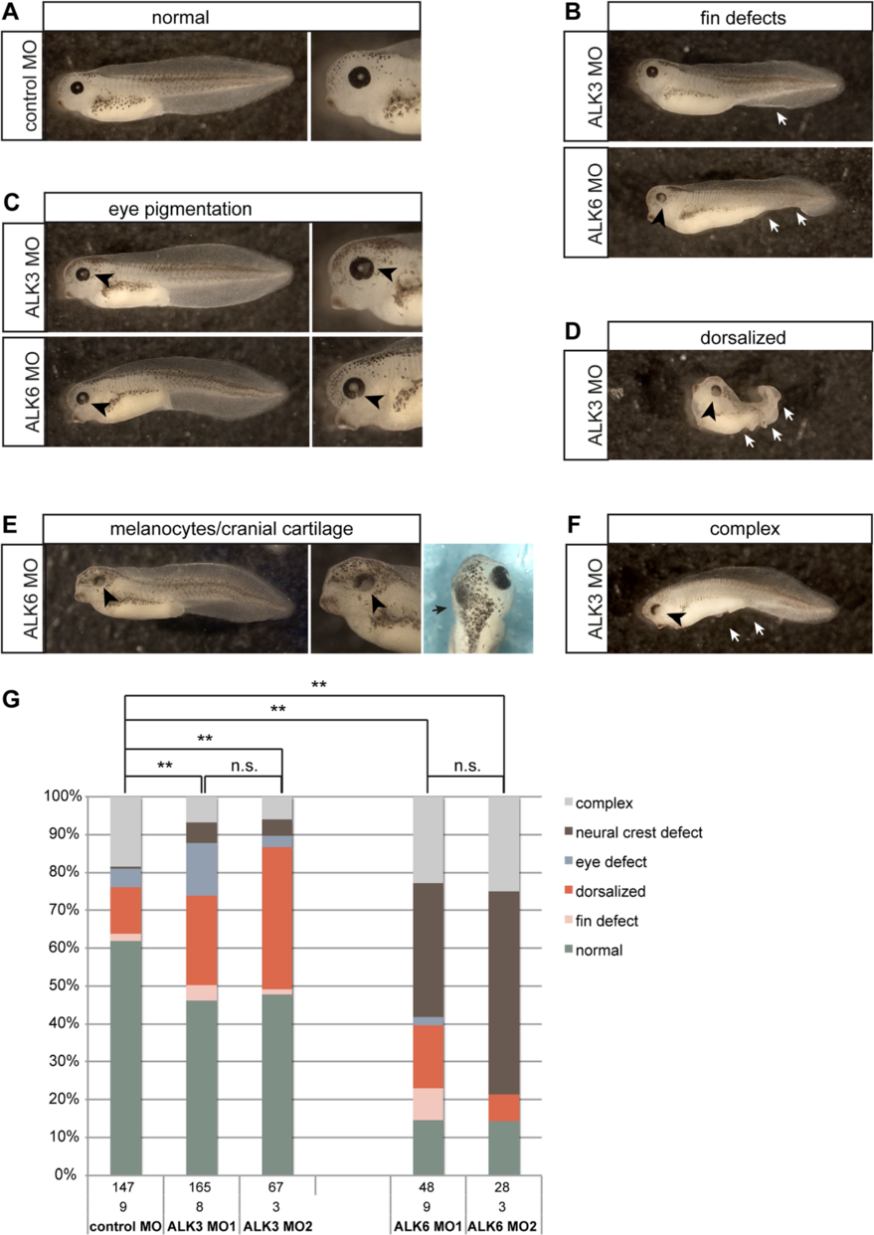
Phenotypes of ALK3 and ALK6 morphant embryos. Embryos were injected with 0.8 pmol antisense morpholino in both blastomeres at the 2-cell stage as indicated and cultured till NF stage 41. **a** Normal NF stage 41 embryos after injection of an unrelated control Morpholino oligonucleotide (control MO). **b** Embryos injected with either ALK3 or ALK6 translation blocking Morpholino oligonucleotides (ALK3 MO and ALK6 MO respectively) showing defects in the development of ventral fins. **c** In the retina of a subset of either ALK3 MO or ALK6 MO injected embryos un- or less pigmented patches were observed. **d** Dorsalized ALK3 morphant embryo. **e** ALK6 morphant embryo showing increased pigmentation of the head and narrower gills. **f** In most cases ALK3 or ALK6 morphant embryos exhibited complex phenotypes with defects in the ventral fin, retina pigmentation, smaller or absent eyes, aberrant pigmentation and loss of cranial cartilage. Black arrowheads indicate eye phenotypes, white arrows missing ventral structures and black arrows mark the cranial cartilage. **g** Phenotype frequencies from at least three independent experiments are summarized in the graph. Differences between indicated groups were analyzed using the *Χ* ^2^ test. Statistically significant deviations (*p*-value < 0.01) are indicated by double asterisks; n.s.: not significant

Compared to control embryos (Fig. 2a), we observed a range of phenotypes in the ALK3 or ALK6 morphant embryos that reached tadpole stage. The weakest phenotypes showed defects or partial loss of the ventral fin (Fig. 2b) or defective pigmentation of the retina (Fig. 2c). Stronger phenotypes included anterio-dorsalized embryos with a progressive loss of ventral and posterior structures (Fig. 2d). A subset of embryos showed increased pigmentation of the head and less protruding branchial arches, indicating that neural crest development was affected (Fig. 2e). This phenotype was further investigated and the results are shown in Figs. 5 and 6. In addition, we observed complex phenotypes that affect multiple structures (Fig. 2f). Dorsalized and eye phenotypes were most frequent in ALK3 morphant embryos and no significant difference was observed between ALK3 MO1 and ALK3 MO2. By contrast, knock-down of ALK6 predominantly resulted in neural crest and pigmentation phenotypes. Although ALK6 MO2 appeared to induce the phenotype with higher frequency, the difference between ALK6 MO1 and ALK6 MO2 was statistically not significant (Fig. 2g).

Homozygous deletion of *alk3* in mice is early embryonic lethal [65]. Because morpholino oligonucleotide injections in two-cell stage *Xenopus* embryos did not deplete maternal protein, early lethality could not be expected. Homozygous a*lk6* knock-out mice are viable and show moderate skeletal phenotypes [66]. The same study suggested overlapping and redundant functions of ALK6 with other BMP receptors in mouse development. Since the knock-down of ALK6 in *Xenopus* embryos resulted in higher lethality and equally severe, albeit different phenotypes as compared to the ALK3 knock-down, less functional overlap of these two type 1 BMP receptors could be assumed in *Xenopus laevis*.

Compound phenotypes in the surviving embryos were expected, based on the complex expression patterns of *alk3* and *alk6*, and are comparable to phenotypes reported for *alk3* or *alk6* loss-of-function in other vertebrates. The role of both, ALK3 and ALK6 in the development of the retina and lens is well established in mouse and chick embryos [67–69]. In addition, defects in brain and cranio-facial development as well as cartilage and bone formation have been described [66, 68–70]. Likewise, we observed defects in the forebrain region, smaller or absent eyes, which indicates that the developmental functions of ALK3 and ALK6 are conserved within vertebrates to a considerable extent. In addition, we observed aberrant pigmentation and cranio-facial deformation, indicating defects in the development of neural crest derived melanocytes and cartilage [71]. Interestingly and most notably, these phenotypes have been predominantly observed in ALK6 morphants, supporting a specific role of ALK6 in the development of the neural crest.

By contrast, we observed predominantly dorsalized phenotypes in ALK3-deficient embryos, which is in agreement with blastula-stage expression of ALK3 and its assumed role in early dorso-ventral patterning. Dorsalized phenotypes were also observed in ALK6 morphant embryos although with lower frequency. The weakest phenotype affecting ventral structures were defects in the ventral fin, development of which depends on BMP signaling. In zebrafish, inactivating or dominant-negative mutations in BMP pathway genes, such as *bmp2b*, *smad5* or *tolloid,* result in the loss of the ventral fin [72, 73]. In *Xenopus*, formation of the ventral fin is similarly dependent on the ventral-most portion of the mesoderm [74] which is specified by BMP signaling. Interestingly, in zebrafish embryos, loss of the ventral fin has been attributed to ALK8, which is most closely related to ALK2 in other vertebrates [75, 76].

The higher frequency of dorsalized embryos in ALK3 morphant embryos, as compared to the ALK6 knock-down was consistent with the expression of these type I BMP receptors in pre-gastrula stage embryos. We already detected *alk3* RNA in early blastula-stage embryos, whereas *alk6* expression remained very low until late gastrula (NF stage 12, Fig. 1). Together these results support a major role of ALK3 and a minor contribution of ALK6 in transducing ventralizing BMP signals in early embryonic development.

### Both type I BMP receptors contribute to dorso-ventral patterning

In order to confirm that the observed dorsalization phenotype in ALK3 and ALK6 morphant embryos was related to a role in early dorso-ventral axis specification, we next analyzed the influence of single and double knock-downs on the expression of the dorsally expressed organizer genes *chordin*, *admp* and *goosecoid (gsc)* [49, 77, 78] as well as the ventral marker genes *bmp4*, *vent1* and *sizzled* [7, 79, 80] in early gastrula stage embryos.

**Fig. 3:**
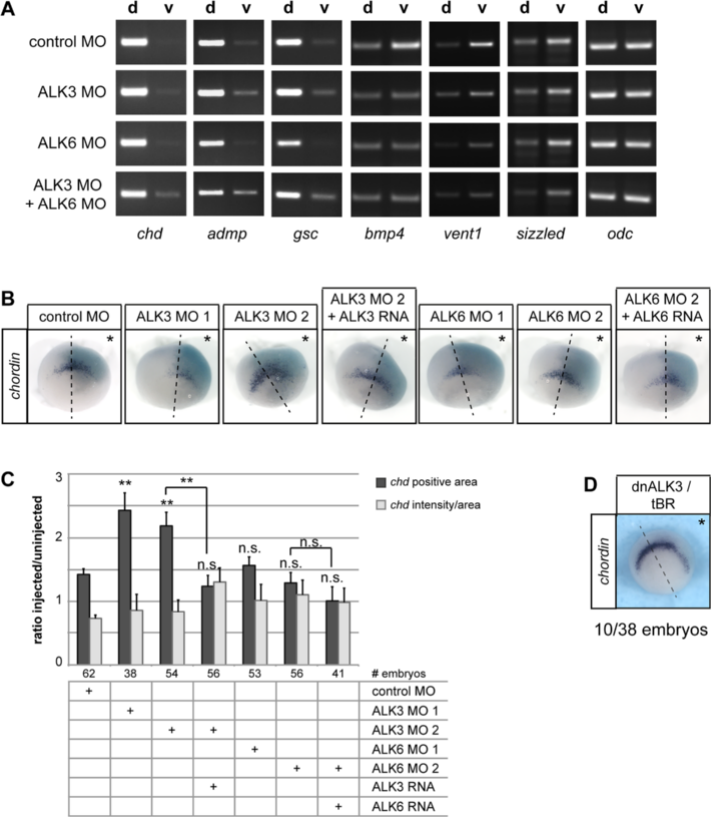
ALK3 and ALK6 are required for dorso-ventral patterning. **a** 2-cell-stage embryos were injected with 1.6 pmol control MO, ALK3 MO or ALK6 MO in both blastomeres as indicated, cultured till stage 10.5 and bisected into dorsal (d) and ventral (v) halves. Expression of the organizer genes *chordin* (*chd*), *admp* and *goosecoid* (*gsc*) and the ventral genes *bmp4*, *vent1* and *sizzled* was analyzed by RT-PCR. The images show one set out of three independent experiments. (**b-g**) Embryos were injected with Fluorescein-labeled dextran as lineage tracer and antisense Morpholino oligonucleotides as indicated into one blastomere at the 2-cell-stage. For rescue experiments, 50 pg of *alk3* RNA or *alk6* RNA were co-injected. The embryos were cultured till stage 10.5 and *chordin* expression was visualized by whole-mount in situ hybridization. **b** Representative examples of phenotypes injected as indicated are shown. Asterisks indicate the injected side, dashed lines mark the dorsal midline as determined by the lineage tracer. **c** The *chordin*-positive area and staining intensity have been measured on the injected and uninjected side of each embryo. The graph displays the average ratio of area size between injected and uninjected side (+SEM) as well as the mean intensity/area (+SEM) from three independent experiments. Differences of area ratios between injection groups have been analyzed using a separate variance *t*-test and statistically significant deviations are indicated by double asterisks (*p*-value < 0.01); n.s.: not significant. The average intensity/area did not change significantly. **d** For comparison, an embryo injected with 50 pg RNA encoding dnALK3 is shown. In 10 out of 38 embryos dnALK3 induced strongly expanded or ectopic *chordin* expression

The transcripts of *chordin*, *admp* and *goosecoid* were exclusively detected in the dorsal halves of control embryos (Fig. 3a). In ALK3 morphants, these dorsal genes were also detected in the ventral halves of the embryos. This effect was weakest for *chordin*, but clearly detectable for *admp* and *goosecoid*. By contrast, in ALK6 morphant embryos the dorsal expression of *admp* and *goosecoid* appeared to be slightly weaker and no expression was detected in the ventral half. In the double morphant embryos, all three dorsal genes were clearly detected in the ventral hemisphere (Fig. 3a). The inverse was observed when we investigated expression levels of the ventrally expressed genes *bmp4*, *vent1* and *sizzled*. Expression of *bmp4* and *vent1* was lower in the ventral halves of ALK3, ALK6 or double morphant embryos. When analyzing *sizzled* expression a detectably weaker expression was only observed in double morphant embryos, whereas single knock-down of either ALK3 or ALK6 had no effect.

Although this experiment was not quantitative, it suggested that knock-down of ALK3 and double knock-down of ALK3 and ALK6 might result in an expansion of the organizer domain into the ventral half of the embryo, which would lead to a dorsalized phenotype. Dorsalization was indeed observed predominantly in ALK3 morphant embryos (see Fig. 2). Overall, these results indicated that both ALK3 and ALK6 play a role in dorso-ventral patterning and contribute to the expression of ventral genes, as demonstrated by the pronounced downregulation of *bmp4*, *vent1* and *sizzled* in double morphant embryos.

In order to further investigate the effect of ALK3 or ALK6 depletion on the organizer, we performed whole mount in situ hybridizations using a *chordin* antisense probe in single-side injected embryos. Consistent with the aforementioned RT-PCR results, the area containing *chordin*-positive cells was enlarged by 50–70 % in ALK3 morphant embryos injected with either ALK3 MO 1 or ALK3 MO 2, while the average staining intensity for *chordin* relative to the expression area remained unchanged (Fig. 3b and c). This expansion of the *chordin*-expressing area was reverted by co-injection of *alk3* RNA. By contrast, depletion of ALK6 did not significantly affect the distribution of *chordin*-expression or the relative staining intensity (Fig. 3b and c). Although ALK3 knock-down significantly expanded the *chordin*-positive area, this effect was mild when compared to the overexpression of a dominant-negative mutant of ALK3 (dnALK3/tBR, [81, 82]). DnALK3 induced strongly expanded and ectopic *chordin* expression in the majority of embryos (Fig. 3d). By contrast, we did not observe ectopic *chordin* expression in ALK3 morphant embryos. This discrepancy is likely due to the presence of maternal protein and possibly a contribution of additional type I receptors, such as ALK2 or ALK4 [41, 42] to the early BMP signal. Both ALK2 and ALK4 are expressed in early *Xenopus* embryos (see Additional file 3: Figure S3) and their activation by BMP ligands would also be inhibited by dnALK3 but not by ALK3 knock-down.

**Fig. 4:**
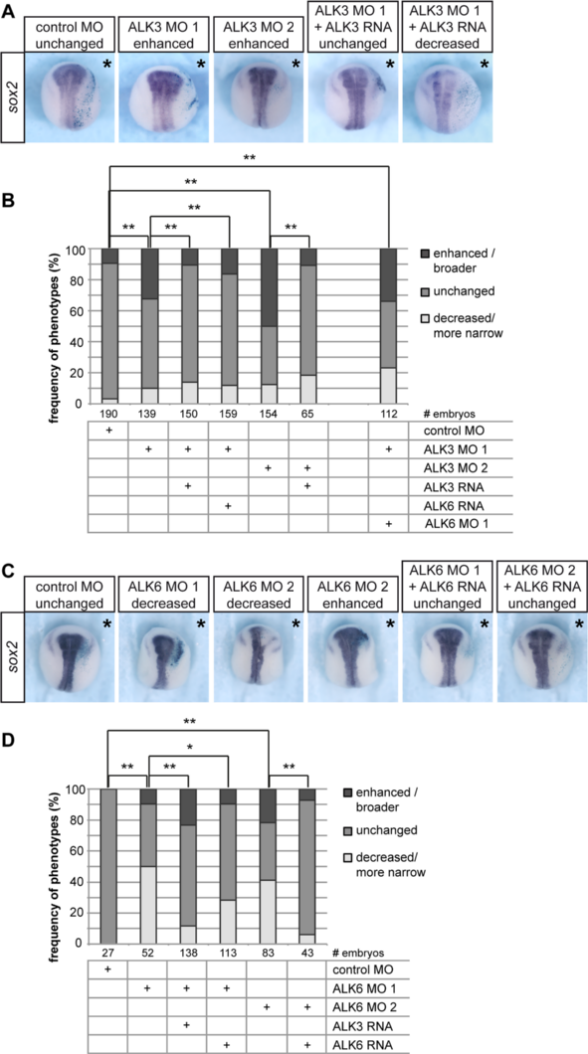
Ectopic ALK6 can substitute ALK3 knock-down in dorso-ventral patterning. Embryos were injected laterally in one dorsal blastomere at the 4-cell stage with 0.8 pg of ALK3 or ALK6 MOs and, for rescue experiments, co-injected with 50 pg of *alk3* RNA or *alk6* RNA. A *lacZ* plasmid was co-injected to identify the injected side, embryos were cultured till stage 18 and stained for LacZ. The neuroectoderm was visualized by in situ hybridization against the pan-neural marker gene *sox2*. Embryos were categorized according to expanded or reduced *sox2* expression and differences between indicated groups were analyzed using the *Χ* ^2^ test. Statistically significant deviations (*p*-value < 0.01) are indicated by double asterisks; n.s.: not significant. **a** Representative images of ALK3 morphant embryos and the corresponding rescue experiments are shown. Embryos were injected as indicated; asterisks indicate the injected side. **b** Results from at least three independent experiments are summarized in the graph. **c** Representative images of ALK6 morphant embryos and the corresponding rescue experiments are shown. Embryos were injected as indicated; asterisks indicate the injected side. **d** Results from at least three independent experiments are summarized in the graph

We next confirmed that the upregulation of dorsal genes in ALK3 morphant embryos observed at early gastrula stages indeed results in an expansion of dorsal tissues; therefore, we analyzed the influence of ALK3 knock-down on neural induction. Consistent with our previous results, single knock-down of ALK3 was sufficient to induce mild broadening of the neural plate and expression of the pan-neural marker *sox2* (Fig. 4a, b). This ALK3 MO 1 phenotype was rescued by co-injection of either morpholino-insensitve ALK3 RNA or ALK6 RNA, demonstrating the specificity of this knock-down phenotype and functional redundancy of ALK3 and ALK6 (see also Additional file 7: Figure S7). Bringing back ALK3 also resulted in a downregulation of *sox2* in roughly 15 % of the embryos, which indicated an over-compensation of the morphant phenotype. Comparable results were obtained using ALK3 MO 2; the size of the *sox2*-positive area was increased by 12.5 ± 2.6 % by injection of ALK3 MO 1 and by 11 ± 3.1 % by ALK3 MO 2 respectively. However, in embryos injected with ALK3 MO 2, broadening of the neural plate occurred more frequently as compared to ALK3 MO 1 (Fig. 4a, b). Double knock-down of ALK3 and ALK6 did not further increase the percentage of embryos showing an enlarged *sox2*-positive area.

We repeated this experiment and targeted the injections to one animal blastomere of eight-cell stage embryos, which should limit the knock-down to the ectoderm and avoid interfering with organizer formation. The outcome was very similar to the results obtained after injection at the four-cell stage (Additional file 6: Figure S6). Ectodermal knock-down of ALK3 by ALK3 MO 1 or ALK3 MO 2 resulted in an increase of the *sox2*-positive area by 14 ± 3.1 and 18 ± 6.9 % respectively. Again, the phenotype was rescued by both *alk3* RNA and *alk6* RNA. These results indicated that in addition to its role in early dorso-ventral specification, ALK3 also influenced ectodermal patterning.

Overall, we have demonstrated a role for ALK3 in the specification of ventral cells fates and ectodermal patterning. However, we observed only moderate dorsalization even after ALK3/6 double knock-down. Our data are consistent with a comparable study in zebrafish in which the double knock-down of ALK3 and ALK6 resulted in only weakly dorsalized phenotypes [56]. Morpholino mediated knock-down did not affect maternal protein, which is possibly a cause for these results. In addition, triple knock-down of the BMP ligands BMP2, BMP4 and BMP7 induced only moderate dorsalization with decreased *sizzled* expression and no effect on *chordin* [50]. The same group showed that strong dorsalization required additional knock-down of ADMP [51]. Knock-down of ALK3 or ALK6 is not expected to affect ADMP activity, because ADMP does not bind to ALK3 or ALK6, but to ALK2 [51]. ALK3 and ALK6 have been identified as receptors for BMP2, BMP4 and BMP7 in multiple studies [34, 64, 83]. Both ALK3 and ALK6 form receptor complexes with BMPRII [31, 36] and BMP2 as well as BMP7 signal via heteromeric receptor complexes comprising different type I receptors in dorso-ventral patterning in zebrafish [84]. Therefore the highly similar effect of BMP2/4/7 knock-down [50] and ALK3/6 knockdown (this study) support the conclusion that ALK3 and ALK6 predominantly act as receptors for BMP2, BMP4 and BMP7 ligands in early dorso-ventral patterning.

Moreover, we observed that ALK3 knock-down is sufficient to expand the expression territory of organizer genes in early gastrula stage embryos, indicating that ALK3 is the dominant type I receptor in the BMP activity gradient that antagonizes expression of organizer genes. Such a bigger role for ALK3 as compared to ALK6 is in agreement with higher expression levels of ALK3 in pre- and early gastrula stages.

We also analyzed *sox2* expression in ALK6 morphant embryos. Interestingly, injection of ALK6 MO 1 or ALK6 MO 2 into one dorsal blastomere of four-cell stage embryos resulted in an anterior downregulation of *sox2* on the injected side at neurula stages (Fig. 4c and d). The size of the *sox2*-positive area was reduced by 10 ± 2.1 and 17 ± 3.1 % in embryos injected with ALK6 MO 1 and ALK6 MO 2, respectively. This phenotype was rescued by co-injection of either *alk3* RNA or *alk6* RNA (see also Additional file 7: Figure S7), which is contradictory to the results shown above that demonstrate a role of ALK3 in ventral specification and antagonizing neural induction. An ALK3 gain-of-function would be expected to inhibit neural induction and thus reduce the size of the *sox2* expression area. In addition, these results overall are contradictory to the theory that BMP activity antagonizes neural induction [6]. Moreover, we did not observe a significant effect of ALK6 knock-down on the size of the organizer as determined by the *chordin*-positive area at early gastrula (Fig. 3b and c). Taking into account that *alk6* expression is very low during gastrulation but upregulated in the anterior neural plate and neural plate border at late gastrula and early neurula stages, this decrease of *sox2* is possibly not caused by alterations of the early BMP gradient. Instead, a role of ALK6 in the development or maintenance of anterior neural tissue after neural induction might be hypothesized, however, the nature and mechanism of such a putative function remains to be investigated.

### ALK6 is required for neural crest specification

The ALK6 morphant phenotype with embryos showing a marked increase of pigment cells, and in some cases a dramatic loss of cranial cartilage, indicated defects in neural crest development (see Fig. 2). Since ALK6 is specifically upregulated in the neural crest during neurulation (Fig. 1a), we analyzed its role in neural crest development in more detail.

**Fig. 5:**
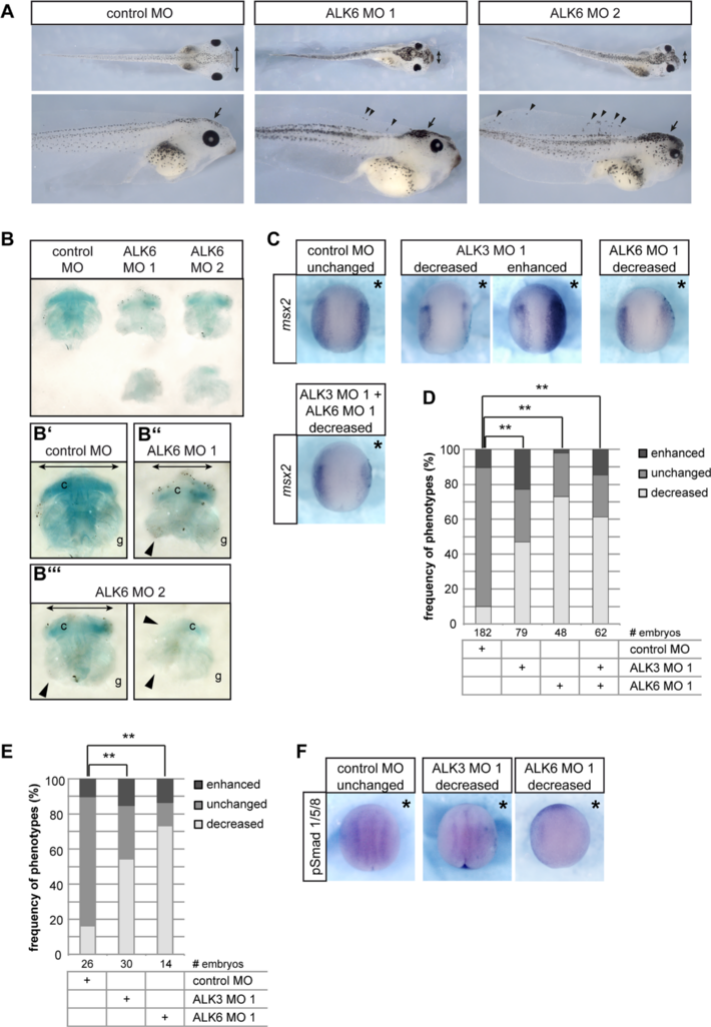
ALK6 is required for neural crest development. **a** When compared to control MO injected embryos, ALK6 morphant embryos showed increased pigmentation of the head (arrow), ectopic melanocytes in the dorsal fin (arrowheads) and narrower heads reflected by less lateral protrusion of the gills and smaller distance between the eyes (double arrow). **b** Cranial cartilage from embryos injected as indicated was stained with Alcian Blue dye. Cartilage from ALK6 morphant embryos generally showed weaker staining, was smaller (double arrows in B’, B” and B”’) and showed partial or complete loss of cartilage elements (arrowheads in B’, B” and B”’) of the gills (g) or ceratohyal cartilage (c). The images in A and B show representative examples from one out of three independent experiments. **c** Embryos were injected with 1.6 pmol morpholino in one animal-dorsal blastomere at the 8-cell stage as indicated. A *lacZ* plasmid was co-injected to identify the injected side, embryos were cultured till stage 13 and stained for LacZ. Expression of *msx2* was analyzed by whole-mount in situ hybridization. Representative images of embryos injected as indicated are shown. Asterisks indicate the injected side. The results from three independent experiments are summarized in the graph (**d**). Active BMP signaling at the neural plate border was shown by whole-mount immunostaining for phosphorylated Smad 1/5/8 (pSmad 1/5/8). **e** The graph shows a summary of pSmad1/5/8 staining results from two independent experiments. In (**f**) representative images of embryos injected with control MO, ALK3 MO 1 or ALK6 MO 1 are shown. Embryos were categorized according to decreased or enhanced *msx2* expression or pSmad 1/5/8 staining respectively and differences between indicated groups were analyzed using the *Χ* ^2^ test. Statistically significant deviations (*p*-value < 0.01) are indicated by double asterisks; n.s.: not significant

ALK6 morphant embryos frequently showed an increased number of melanocytes in the dorsal head region, which was in most cases accompanied by deformed branchial arches (Fig. 1). Closer examination of these embryos revealed aberrant localization of melanocytes in both ALK6 MO 1 and ALK6 MO 2 injected embryos. Many melanocytes were found in the dorsal head region, whereas the flank in most embryos was less pigmented. In addition, we observed ectopic melanocytes in the dorsal fin (Fig. 5a), indicating aberrant specification or migration of neural crest derived melanocyte precursors.

Embryos showing aberrant pigmentation were subjected to Alcian Blue staining of the cranial cartilage. All ALK6 morphant embryos showed fainter cartilage staining, indicating incomplete cartilage differentiation. In addition, we observed a range of defects in the cranial cartilage. In milder cases, parts of the gills were missing and the ceratohyal cartilage was smaller or deformed; overall, the cartilage elements present were only faintly stained. In the most severe cases, only tiny, unstructured remnants of cartilage were found (Fig. 5b). The requirement of ALK6 in chondrogenic differentiation of the cranial cartilage, as indicated by reduced Alcian Blue staining, is consistent with the role of ALK6 in cartilage and bone formation in the mouse [66, 85]. Moreover, the partial or complete loss of cranial cartilage elements (Fig. 5b) together with the prominent expression of *alk6* in pre-migratory and migrating neural crest cells (Fig. 1a) indicated an additional role in early neural crest development.

Neural crest development begins in the dorso-lateral marginal zone of early gastrula embryos in *Xenopus*. In this region attenuated BMP activity combined with active Wnt/β-Catenin signaling specifies neural crest precursors [14, 86]. After gastrulation neural crest precursors are located at the neural plate border and identified by the expression of a specific set of transcription factors, including *msx1* and *msx2,* that induce further neural crest development [59, reviewed in 87].

To address the question of whether the neural crest is not induced in ALK6 morphants or if neural crest cells get lost during pre-migratory development, we analyzed the expression of *msx2* in the neural plate border at late gastrula stages. Both ALK3 and ALK6 loss-of-function resulted in a downregulation of *msx2*, although 23 % of ALK3 morphant embryos showed an increase of *msx2* (Fig. 5c and d). In the ALK6 morphants, no increased *msx2* expression was observed, but more than 70 % of embryos showed a decrease or loss of *msx2* expression (Fig. 5d), thus demonstrating further that particularly ALK6 plays a crucial role in neural crest development. Interestingly, the frequency of increased and decreased *msx2* expression in double morphant embryos represented the average of phenotype frequencies that were observed in the single knock-downs (Fig. 5d), indicating that ALK3 and ALK6 affect *msx2* expression independently of each other.

It has been shown that reducing BMP activity in blastula or gastrula stages results in a shift and expansion of the neural plate border area as well as upregulation of neural crest markers [88]. Such expansion of the *msx2* expression area was only observed in approximately one quarter of ALK3 morphant embryos, which is consistent with the role of ALK3 in dorso-ventral patterning discussed above. On the other hand, *msx1* and *msx2* are regulated by BMP signaling, and *msx2* has been identified as a direct BMP target [13, 60, 89]. The observed downregulation of *msx2* in ALK3 and ALK6 morphant embryos coincided with the upregulation of both type I receptors at the neural plate border in late gastrulation (see Fig. 1a); together, these observations strongly suggested a role for ALK3 and ALK6 in this Smad-dependent transcriptional regulation.

To confirm that both receptors signal to Smad at the neural plate border, we probed NF stage 13 embryos for the presence of active phosphorylated Smad1/5/8 (pSmad1/5/8) using a phospho-specific antibody. We observed a narrow stripe of pSmad1/5/8 positive cells along the neural plate border. This local activation of BMP signaling was detectable from NF stage 12.5 onward, again coinciding with the upregulation of ALK6 as well as ALK3, albeit to a lesser extent, at the neural plate border. After injection of ALK3 MO, we detected a decrease of pSmad 1/5/8 at the neural plate border in approximately 50 % of the embryos. The effect was again more pronounced in ALK6 morphants (Fig. 5e and f), which showed a reduction of pSmad 1/5/8 in over 70 % of the embryos. This result demonstrated that both ALK3 and ALK6 activate Smad in neural plate border cells. Moreover, pSmad data were highly similar to the *msx2* expression data (Fig. 5d and f), which is consistent with *msx2* being a direct target gene of BMP signaling [60]. Therefore it is conceivable that both ALK3 and ALK6 contribute to the activation of Smad at the neural plate border, thereby upregulating BMP-responsive neural plate border genes such as *msx1* and *msx2*. According to current models of neural crest development in *Xenopus*, neural crest precursors are first specified at early gastrula stages by the combined input of low BMP and low Wnt/β-Catenin signaling [14, 90]. During gastrulation, cells located lateral of the future neural plate receive Wnt/β-Catenin, FGF, Notch and BMP signals that induce the expression of neural plate border specifying or pre-patterning genes such as *gbx2*, *pax3*, *zic1*, *msx1* and the BMP target gene *msx2* [13, 58–60, 87, 91–93]. These neural plate border factors govern further induction of neural crest cells and the expression of genes required for proliferation, survival and migration of the neural crest, including *ap2α*, *foxd3*, *sox8*, *sox9*, *sox10*, *slug*, *snail* and *twist* [58, reviewed in: 59, 87, 94–99].

**Fig. 6:**
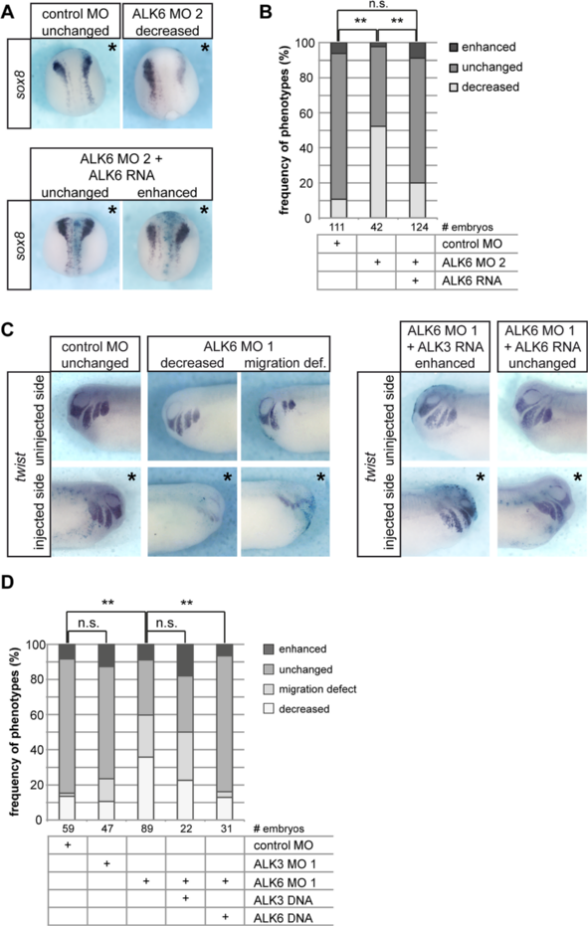
Analysis of *sox8* and *twist* expression in ALK6 morphant embryos. Embryos were injected with 1.6 pmol morpholino targeted animally in one dorsal blastomere at the 4-cell stage. A *lacZ* plasmid was co-injected to identify the injected side, embryos were cultured till stage 18 or 24 and stained for LacZ. The neural crest was visualized by in situ hybridization against *sox8* at stage 18 and against *twist* at stage 24. Differences between indicated groups were analyzed using the *Χ* ^2^ test. Statistically significant deviations (*p*-value < 0.01) are indicated by double asterisks; n.s.: not significant. **a** Representative images of embryos hybridized against *sox8* and injected as indicated are shown. The ALK6 MO phenotype was rescued by co-injection of 50 pg *alk6* RNA. Asterisks indicate the injected side. The results from three independent experiments are summarized in the graph (**b**). **c** Representative images of the uninjected and injected sides of embryos of stage 24 embryos hybridized against *twist* are shown. Embryos were injected with 1.6 pmol of ALK6 MO 1; for rescue experiments 25 pg of *alk3* plasmid DNA or *alk6* plasmid DNA were co-injected. The results from three independent experiments are summarized in the graph (**d**)

Consistent with a role of ALK6 in neural plate border specification, *sox8* was severely downregulated in ALK6 depleted embryos at neurula stages (Fig. 6a and b). We observed decreased expression of *sox8* in approximately 50 % of all embryos injected with ALK6 MO2. Co-injection of MO-insensitive *alk6* RNA fully rescued *sox8* expression and further supported a role of ALK6 in neural crest induction (Fig. 6a and b). Interestingly, the frequency of the neural crest induction phenotype dropped from over 70 % of embryos showing decreased *msx2* expression at NF stage 13 to 50 % embryos with decreased *sox8* expression at NF stage 18. This decline of phenotype frequency indicated a partial regeneration of neural crest cells; such regenerative potential has also been observed in chick embryos after removal of neural crest cells [100]. In addition, proliferation of pre-migratory neural crest cells could contribute to the observed partial recovery of the neural crest population between NF stage 13 and NF stage 18.

We next visualized the migrating neural crest in NF stage 26 embryos by in situ hybridization for *twist* expression. ALK6 MO 1 injection induced a downregulation of *twist* in 36 % of the embryos (Fig. 6c and d). This further decline in frequency of the “decreased” neural crest marker gene phenotype supports the assumption that the neural crest population is partially recovering in ALK6 morphant embryos. By contrast, single knock-down of ALK3 resulted in less than 15 % of induction defects (Fig. 6d) as compared to 45 % at NF stage 13 (Fig. 5d). This might simply indicate a delayed specification of the neural plate border in ALK3 depleted embryos, where ALK3 function could be compensated albeit belatedly by redundant proteins, such as ALK6, during later neural crest development. Notably, ALK6 knock-down was not fully compensated until tadpole stages, which suggests a partially non-redundant role of ALK6 in post-induction neural crest development. Such an additional role of ALK6 is further supported by the occurrence of impaired neural crest migration in 24 % of ALK6 morphant embryos that was not observed in ALK3 MO-injected embryos (Fig. 6c and d). However, it cannot be concluded whether ALK6 itself plays a direct role in neural crest migration or if the observed migration defects are secondary effects of impaired neural crest specification in these embryos.

To rescue the ALK6 MO phenotype without interfering with dorso-ventral patterning, we co-injected plasmids encoding ALK3 or ALK6 that are expressed only when zygotic transcription is initiated after the mid-blastula transition. The neural crest phenotype in ALK6 morphants was not rescued by co-injection of *alk3* plasmid DNA, although an upregulation of *twist* was observed in a low percentage of embryos when ALK3 was overexpressed in the ALK6 morphant background (Fig. 6c and d). By contrast, ALK6 MO was fully rescued by co-injection of *alk6* plasmid DNA (Fig. 6c and d), which again confirmed a specific, non-redundant function of ALK6 in the neural crest. Injection of plasmid DNA typically results in a mosaic expression of the gene. Assuming that the ALK6 knock-down phenotype could be compensated partially by expansion of the residual neural crest progenitor population, the plasmid would restore ALK6 in a subset of cells and thereby increase this progenitor population sufficiently to allow full regeneration of the neural crest. Notably, maintenance of the neural crest fate during neurulation requires secreted growth factors that are derived from adjacent tissues including BMPs from the non-neural ectoderm and Wnt signals from the underlying paraxial mesoderm [14, 61, 90]. Therefore, ALK6 function could also be indirect via a secreted factor. In this case, restoring ALK6 function in a mosaic pattern by plasmid DNA injection would also be sufficient to fully rescue the phenotype because the secreted protein would act on all receiving cells within its range of action.

Altogether, these results demonstrate a role of ALK3 and ALK6 in the early specification of neural crest precursors whereas exclusively ALK6 is required for neural crest maintenance and migration in post-gastrula stages.

In this study, we have demonstrated that ALK3 and ALK6 are required for early neural crest precursor specification during gastrulation, although likely to be independent from each other. Consistent with a role of both receptors in early BMP-dependent dorso-ventral patterning of the embryo as shown and discussed above, it is conceivable that ALK3 and ALK6 signaling first contributes to positioning of the neural plate border and then mediates the moderate activation of BMP signaling in cells lateral of the neural plate that leads to the expression of neural plate border genes such as *msx2*. At late gastrula and early neurula stages, ALK3 is expressed in this region, and ALK6 expression is upregulated in the anterior neural plate and at the neural plate border from NF stage 12 onwards. This locally elevated expression correlates temporally and spatially with a local activation of Smad, and we have demonstrated that once again both type I receptors play a role in this process. However, we also observed that specifically ALK6, but not ALK3, plays an essential role in post-gastrula neural crest development.

The transition from cell fate specification at the neural plate border to maintenance of neural crest identity and further development takes place in early neurula stages. Based on expression patterns and functional analyses, we propose that a switch from an early, cooperative and redundant function of ALK3 and ALK6 in neural crest specification towards a highly specific function of ALK6 in neural crest maintenance and migration takes place during this phase. This could be related to a change in BMP ligands that are available. During early embryonic development, BMP2, BMP4 and BMP7 are expressed and required for dorso-ventral patterning, and all three ligands can signal via ALK3 or ALK6 or heteromeric receptor complexes [34, 64, 84]. A recent study in zebrafish showed that in early neurulation two distinct domains of active BMP signaling demarcate the borders between epidermis, pre-placodal ectoderm and the neural crest; it also showed that this local BMP activation depends on GDF6, also known as BMP13 [101]. In *Xenopus*, *gdf6* is expressed in a single stripe at the neural plate border from late gastrula stage until late neurula stages [102], overlapping with ALK6 expression as shown in Fig. 1a. Consistently, we detected only one stripe of pSmad1/5/8 along the neural plate border at NF stage 13. Despite this difference, which might be due to temporal divergence of neural crest and placodal specification between zebrafish and *Xenopus*, it is likely that GDF6 mediated local activation of BMP signaling in the neural crest is conserved in both species. GDF6 signals via ALK3 and ALK6 but displays a preference towards ALK6 [42]. We have demonstrated herein that in *Xenopus* the transition from cell fate specification to maintenance of neural crest identity is accompanied by a shift in type I receptor requirement towards ALK6. Therefore we propose that post-gastrula neural crest development specifically requires ALK6 to mediate GDF6 signaling, a hypothesis that needs to be further investigated in the future.

## Conclusion

Here we have demonstrated overlapping and non-redundant functions of the type I BMP receptors ALK3 and ALK6 in early *Xenopus* development. Both contribute to BMP dependent ventral identity, but ALK6 is additionally required for gene expression and size of the dorsal organizer in *Xenopus* embryos. These results show that despite overlapping expression patterns and activation of the same Smad signaling pathway, ALK3 and ALK6 exert temporally and spatially specific, diverging functions; indeed, they are important factors in the regulatory network that controls patterning of early embryos and the ectoderm.

In addition, we have demonstrated a highly specific expression of ALK6 in the neural crest and an exclusive requirement of ALK6 in neural crest induction and development. ALK6 likely acts a receptor for GDF6 in the neural crest and mediates neural crest development via an evolutionary conserved mechanism in the transition from neural crest specification to maintenance and further determination of cell fate.

## Methods

### Morpholinos, plasmids and antibodies

ALK3 and ALK6 Morpholinos (ALK3 MO 1: TTCTTGAAGAAGTATGCTCGCCTTT; ALK3 MO 2: CAACCAACCCATCCCTTGTGCCGGA; ALK6 MO 1: ACTCCACTCTCTGTTCCTCCTTTGT; ALK6 MO 2: TTGGGAACTTGTCAAACAACCGGCC) and a Control Morpholino (Control MO) were purchased from Gene Tools LLC, USA.

Full length coding sequences for *Xenopus alk3*, *alk6,* and *msx2* were amplified from a stage 11–17 cDNA pool and cloned into the pCRII TA vector (ThermoFisher Scientific, USA). For cloning of the Flag-tagged full-length *Xenopus* ALK3 and ALK6 (ALK3-Flag and ALK6-Flag), the coding sequences of *Xenopus alk3* and *alk6* were subcloned into the EcoRI and XhoI sites of a pCS2+ vector [103] in frame with a Flag-tag. The 5′ UTRs of *alk3* and *alk6* were similarly amplified and cloned into the pCS2+ vector 5′ of an *egfp* coding sequence. The full-length sequence of the *alk6* mRNA has been submitted to Genebank (BankIt1812091 *Xenopus*, KR052160). The in situ probes for *chordin*, *sox2* and *twist* were kind gifts of E. DeRobertis and Doris Wedlich.

The following commercial antibodies were used: rabbit anti-GFP (Abcam, UK) and mouse anti-GAPDH (Proteintech Group, Inc., USA). Secondary antibodies were anti-mouse-Alkaline Phosphatase and anti-rabbit-Alkaline Phosphatase (Cell Signaling Technology, Inc. USA).

### Xenopus laevis embryos and microinjection

*Xenopus* embryos were generated and cultured according to general protocols and staged according to the normal table of Nieuwkoop and Faber [55]. All procedures were performed according to the German animal use and care law (Tierschutzgesetz) and approved by the local authorities and committees (animal care and housing approval: I/39/EE006, Veterinäramt Erlangen; animal experiments approval: 54–2532.2–8/08, German state administration Bavaria/Regierung von Mittelfranken).

RNA for microinjection was prepared using the mMessage mMachine Kit (ThermoFisher Scientific, USA). The following amounts were injected: 500 pg for *alk3–5′-gfp* and *alk6–5′-gfp*, 50 pg of *alk3-flag* and *alk6-flag* RNA; 25 pg of pCS2 *alk3-flag* and pCS2 *alk6-flag* DNA and 100 pg of pCS2*-β-galactosidase* DNA. Knock-down was achieved by injection of 0.8 pmol or 1.6 pmol of ALK3 MO and ALK6 MO as indicated.

Embryos were injected and cultured until they reached the desired stage as indicated.

For subsequent in situ hybridization, single side injections were performed and traced by co-injection of pCS2*-β-galactosidase* DNA. The injected side was visualized by ß-galactosidase staining and in situ hybridizations were carried out, as described by Harland [104], using *chordin* [77], *sox2* [105], *twist* [106]*, msx2, alk3, alk6 or bmpr2* as antisense probes, respectively.

### RT-PCR

Total RNA was extracted from *Xenopus* embryos of the indicated developmental stages (High Pure RNA Isolation Kit, Roche, Germany), reverse transcribed using MMLV reverse transcriptase (New England Biolabs, USA) and the indicated transcripts were amplified from the resulting cDNA using OneTaq Polymerase (New England Biolabs, USA). Primer sequences are provided in Additional file 8: Table S1. For quantification of transcription levels of *alk3* and *alk6*, real-time RT-PCR was carried out using Brilliant III Sybr Green Master Mix and the Agilent AriaMX Real Time PCR system (Agilent Technologies, USA). Transcription levels of *alk3* and *alk6* were calculated using synthetic *alk3* and *alk6* cDNA as external standard.

### Preparation of embryo lysates and western blotting

Embryos were lysed in Lysis buffer (20 mM HEPES pH 7.9, 200 mM KCl, 0.5 mM DTT, 0.5 mM EDTA, 0.5 % NP–40, 20 % Glycerol) supplemented with complete Protease Inhibitor and PhosStop Phosphatase Inhibitor Cocktails (Roche, Germany) at 4 °C. Lysates were cleared at 16,000 × g for 10 min, and the protein concentration was determined using a BCA Assay, according to the manufacturer’s instructions (Applichem, Germany). For Western blotting, proteins were separated in 10 % polyacrylamid gels [107] and transferred to PVDF membranes; the desired proteins were then visualized by incubation with the appropriate antibodies and colorimetric detection using NBT/BCIP.

### Availability of supporting data

Additional data supporting the methods and results of this article are included within the article and its additional files.

## Additional files

Additional file 1: Figure S1. Alignment of ALK6 and phylogenetic analysis of ALK3 and ALK6 protein sequences. (PDF 174 kb) Additional file 2: Figure S2. Temporal and spatial expression pattern of *alk3* and *alk6*. (PDF 1708 kb) Additional file 3: Figure S3. Expression of Activin receptors in dorso-ventrally bisected embryos. (PDF 131 kb) Additional file 4: Figure S4. ALK3 MO and ALK6 MO specificity. (PDF 497 kb) Additional file 5: Figure S5. ALK6 morphant embryos showed a lower survival rate. (PDF 116 kb) Additional file 6: Figure S6. Knock-down of ALK3 in the dorsal ectoderm results in an expansion of neural tissue. (PDF 159 kb) Additional file 7: Figure S7. Phenotypes of ALK3 and ALK6 morphant embryos are rescued with *alk3* or *alk6* RNA. (PDF 361 kb) Additional file 8: Table S1. Primer sequences for RT-PCR and cloning. (PDF 51 kb)
