# Supplementary material for: Differential requirement of bone morphogenetic protein receptors Ia (ALK3) and Ib (ALK6) in early embryonic patterning and neural crest development
Source: BMC Dev Biol. 2016 Jan 19;16:1. doi: 10.1186/s12861-016-0101-5 (PMC4717534; doi:10.1186/s12861-016-0101-5)
Supplement: Additional file 1: Figure S1. — Alignment of ALK6 and phylogenetic analysis of ALK3 and ALK6 protein sequences. (PDF 174 kb) [file 12861_2016_101_MOESM1_ESM.pdf]

A

```
Xenopus laevis        MGWLEERNWQLHVSLIMLLSLHTR--ANLLDNMLLRSSAKLNVDSRKEDTESTAPAPPPKMLSCYCHHHCPEDSFNNTCWTDGYCFTIVEEDESGLPVVT 98
Xenopus tropicalis    MGWMEERNWQLHVSLIMLLSLHTR--ANLLDNMLLRSSAKLNVDSRKEDTESTAPAPPQKMLACYCHHHCPEDSFNNTCWTDGYCFTIVEEDESGLPVVT 98
chicken               --------------------------------MPLLSSSKLSMESRKEDSEGTAPAPPQKKLSCQCHHHCPEDSVNSTCSTDGYCFTIIEEDDSGGHLVT 68
mouse                 --------------------------------MLLRSSGKLNVGTKKEDGESTAPTPRPKILRCKCHHHCPEDSVNNICSTDGYCFTMIEEDDSGMPVVT 68
human                 --------------------------------MLLRSAGKLNVGTKKEDGESTAPTPRPKVLRCKCHHHCPEDSVNNICSTDGYCFTMIEEDDSGLPVVT 68
zebrafish             MDLYRSCLRWLCVLLIGLLGLVEENLANVLDSMLLRNSGK--VEERRETG-SDAPALPQRFLWCYCYHHCPEDSTNNTCRTDGYCFTMVEE-EGGAAVVT 96
                                                    * * .:.*  :  ::*     . **:   : * * *:******* *. * *******::** :.*  :**

Xenopus laevis        SGCLGLEGSDFQCRDTPIPHQRRSIECCTDEDFCNRDLHPTLPPLKKTNFVDGSIHYMALLISVTVCSIILAIILIFCYFRYKRQEARPRYSIGIEQDET 198
Xenopus tropicalis    SGCLGLEGSDFQCRDTPIPHQRRSIECCTDEDFCNRDLHPTLPPLKKPDFVDGSIHYMALLISVTVCSIILAIILIFCYFRYKRQEARPRYSIGIEQDET 198
chicken               KGCLGLEGSDFQCRDTPIPHQRRSIECCTGQDYCNKHLHPTLPPLKNRDFAEGNIHHKALLISVTVCSILLVLIIIFCYFRYKRQEARPRYSIGLEQDET 168
mouse                 SGCLGLEGSDFQCRDTPIPHQRRSIECCTERNECNKDLHPTLPPLKDRDFVDGPIHHKALLISVTVCSLLLVLIILFCYFRYKRQEARPRYSIGLEQDET 168
human                 SGCLGLEGSDFQCRDTPIPHQRRSIECCTERNECNKDLHPTLPPLKNRDFVDGPIHHRALLISVTVCSLLLVLIILFCYFRYKRQETRPRYSIGLEQDET 168
zebrafish             SGCLGLVGSEFQCRDTGNSKQRRALECCTDQDYCNRDLHPTLPPLRTPSYVVGDIHHIALLISVSVCSFILTFIIIFCYFRYKRHELAPRYSLGLHPDES 196
                      .***** **:******  .:***::**** .: **:.********:  .: . * **:******:***::*.:*::********:*  ****:*:. **:

Xenopus laevis        YIPPGELLKDLIEQSQSSGSGSGLPLLVQRTIAKQIQMVKQIGKGRYGEVWMGKWRGEKVAVKVFFTTEEASWFRETEIYQTVLMRHENILGFIAADIKG 298
Xenopus tropicalis    YIPPGESLKDLIEQSQSSGSGSGLPLLVQRTIAKQIQMVKQIGKGRYGEVWMGKWRGEKVAVKVFFTTEEASWFRETEIYQTVLMRHENILGFIAADIKG 298
chicken               YIPPGESLKDLIEQSQSSGSGSGLPLLVQRTIAKQIQMVKQIGKGRYGEVWMGKWRGEKVAVKVFFTTEEASWFRETEIYQTVLMRHENILGFIAADIKG 268
mouse                 YIPPGESLRDLIEQSQSSGSGSGLPLLVQRTIAKQIQMVKQIGKGRYGEVWMGKWRGEKVAVKVFFTTEEASWFRETEIYQTVLMRHENILGFIAADIKG 268
human                 YIPPGESLRDLIEQSQSSGSGSGLPLLVQRTIAKQIQMVKQIGKGRYGEVWMGKWRGEKVAVKVFFTTEEASWFRETEIYQTVLMRHENILGFIAADIKG 268
zebrafish             FIPAGESLRDLIEQSQSSGSGSGLPLLVQRTIAKQIQMVTQIGKGRYGEVWMGRWRGEKVAVKVFFTTEEASWFRETEIYQTVLMRHDNILGFIAADIKG 296
                      :**.** *:******************************.*************:*********************************:************

Xenopus laevis        TGSWTQLYLITDYHENGSLYDYLKSTTLDTKAMLKLAYSTVSGLCHLHTEIFGTKGKPAIAHRDLKSKNILVKKNGTCCIADLGLAVKFISDTNEVDIPP 398
Xenopus tropicalis    TGSWTQLYLITDYHENGSLYDYLKSTTLDTKAMLKLAYSTVSGLCHLHTEIFGTKGKPAIAHRDLKSKNILVKKNGTCCIADLGLAVKFISDTNEVDIPP 398
chicken               TGSWTQLYLITDYHENGSLYDYLKSTTLDTKGMLKLAYSSVSGLCHLHTGIFSTQGKPAIAHRDLKSKNILVKKNGTCCIADLGLAVKFISDTNEVDIPP 368
mouse                 TGSWTQLYLITDYHENGSLYDYLKSTTLDAKSMLKLAYSSVSGLCHLHTEIFSTQGKPAIAHRDLKSKNILVKKNGTCCIADLGLAVKFISDTNEVDIPP 368
human                 TGSWTQLYLITDYHENGSLYDYLKSTTLDAKSMLKLAYSSVSGLCHLHTEIFSTQGKPAIAHRDLKSKNILVKKNGTCCIADLGLAVKFISDTNEVDIPP 368
zebrafish             TGSWTQLYLITDYHENGSLYDYLKCTTLDSRAMLKLAYSSVSGLCHLHTEIFGTQGKPAIAHRDLKSKNILVKRNGACCIADLGLAVKFISDTNEVDIPL 396
                      ************************.****::.*******:********* **.*:******************:**:**********************

Xenopus laevis        NTRVGTKRYMPPEVLDESLNRNHFQSYIMADMYSFGLILWEVARRCISGGILEEYQLPYHDLVPSDPSYEDMRDIVCIRRLRPSFPNRWSSDECLRQMGK 498
Xenopus tropicalis    NTRVGTKRYMPPEVLDESLNRNHFQSYIMADMYSFGLILWEVARRCISGGILEEYQLPYHDVVPSDPSYEDMREIVCIRRFRPSFPNRWSSDECLRQMGK 498
chicken               NTRVGTKRYMPPEVLDESLNRNHFQSYIMADMYSFGLILWEIARRCVSGGIVEEYQLPYHDLVPSDPSYEDMREIVCIKRLRPSFPNRWSSDECLRQMGK 468
mouse                 NTRVGTKRYMPPEVLDESLNRNHFQSYIMADMYSFGLILWEIARRCVSGGIVEEYQLPYHDLVPSDPSYEDMREIVCMKKLRPSFPNRWSSDECLRQMGK 468
human                 NTRVGTKRYMPPEVLDESLNRNHFQSYIMADMYSFGLILWEVARRCVSGGIVEEYQLPYHDLVPSDPSYEDMREIVCIKKLRPSFPNRWSSDECLRQMGK 468
zebrafish             NTRVGTKRFMAPEVLDETLNRNHFQSYIMADMYSFGLILWEISRRCVTGGIVEEYQLPYHDHVPNDPSYEDMREVVCIKRIRPSFPNRWSSDECLRQMGK 496
                      ********:*.******:***********************::***::** *:**********.********::**::::*******************

Xenopus laevis        LMTECWAHNPASRLTALRVKKTLAKMSESQDIKL 532
Xenopus tropicalis    LMTECWAHNPASRLTALRVKKTLAKMSESQDIKL 532
chicken               LMMECWAHNPASRLTALRVKKTLAKMSESQDIKL 502
mouse                 LMTECWAQNPASRLTALRVKKTLAKMSESQDIKL 502
human                 LMTECWAHNPASRLTALRVKKTLAKMSESQDIKL 502
zebrafish             LMTECWAHNPASRLTALRVKKTLAKMSESQDIKV 530
                      ** ****:*************************:
```

B

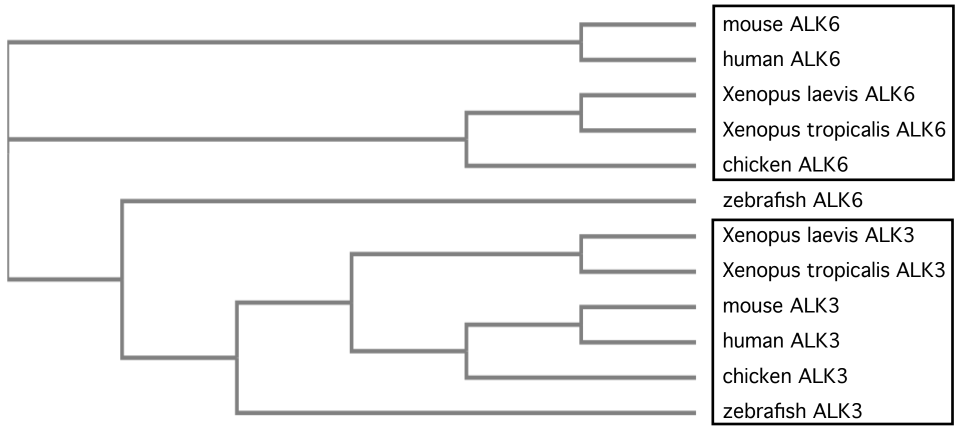

Additional Figure 1. Alignment of ALK6 and phylogenetic analysis of ALK3 and ALK6 protein sequences. The novel alk6 mRNA sequence has been submitted to Genebank (accession number KR052160). (A) Multiple alignment of the translated ALK6 protein sequence to Xenopus tropicalis, chicken, mouse, human and zebrafish ALK6. (B) Phylogenetic tree computed from alignments of Xenopus laevis ALK3 and ALK6 sequences to the Xenopus tropicalis, chicken, mouse, human and zebrafish orthologues. Alignment and phylogenetic tree were calculated using ClustalW2 (Larkin et al. 2007, Li et al. 2015, Saitou and Nei 1987); the corresponding data sets are available from TreeBase at http://purl.org/phylo/treebase/phylows/study/TB2:S18663.
