# Supplementary figures and images for: Differential requirement of bone morphogenetic protein receptors Ia (ALK3) and Ib (ALK6) in early embryonic patterning and neural crest development
Source: BMC Dev Biol. 2016 Jan 19;16:1. doi: 10.1186/s12861-016-0101-5 (PMC4717534; doi:10.1186/s12861-016-0101-5)

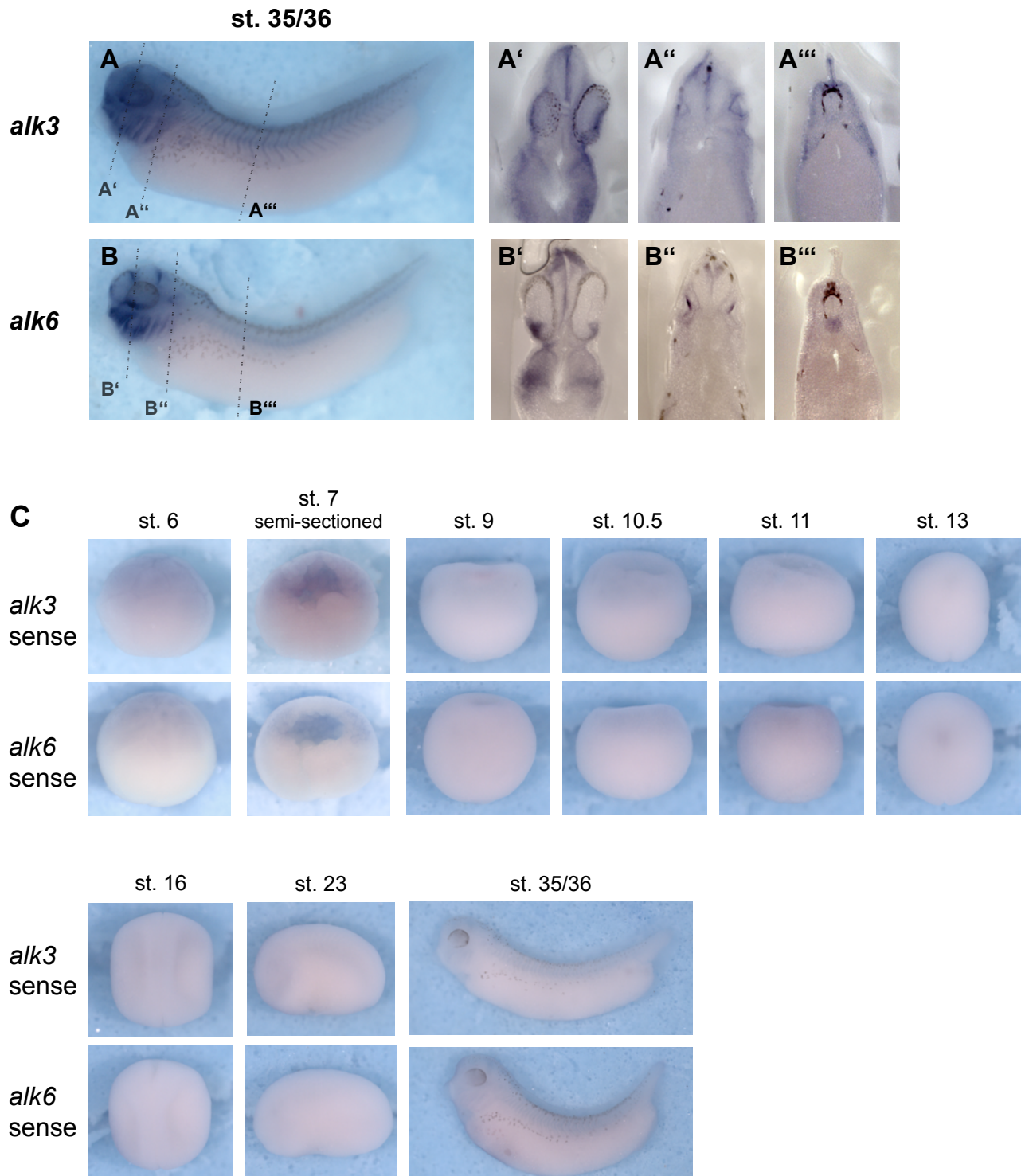

Supplement: Additional file 2: Figure S2. — Temporal and spatial expression pattern of alk3 and alk6. (PDF 1708 kb) [file 12861_2016_101_MOESM2_ESM.pdf]
