# Supplementary material for: Differential requirement of bone morphogenetic protein receptors Ia (ALK3) and Ib (ALK6) in early embryonic patterning and neural crest development
Source: BMC Dev Biol. 2016 Jan 19;16:1. doi: 10.1186/s12861-016-0101-5 (PMC4717534; doi:10.1186/s12861-016-0101-5)
Supplement: Additional file 3: Figure S3. — Expression of Activin receptors in dorso-ventrally bisected embryos. (PDF 131 kb) [file 12861_2016_101_MOESM3_ESM.pdf]

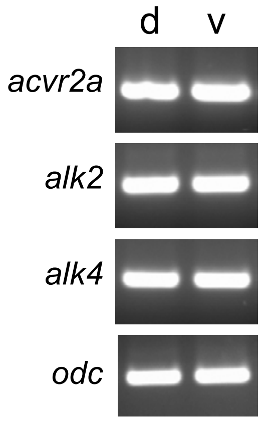

Additional Figure 3. Expression of Activin receptors in dorso-ventrally bisected embryos. Transcripts of activin receptor 2a, alk2 and alk4 were detected in dorsal and ventral halves of NF stage 10.5 embryos by RT-PCR. None showed a dorso-ventrally biased expression.
