# Supplementary material for: Differential requirement of bone morphogenetic protein receptors Ia (ALK3) and Ib (ALK6) in early embryonic patterning and neural crest development
Source: BMC Dev Biol. 2016 Jan 19;16:1. doi: 10.1186/s12861-016-0101-5 (PMC4717534; doi:10.1186/s12861-016-0101-5)
Supplement: Additional file 4: Figure S4. — ALK3 MO and ALK6 MO specificity. (PDF 497 kb) [file 12861_2016_101_MOESM4_ESM.pdf]

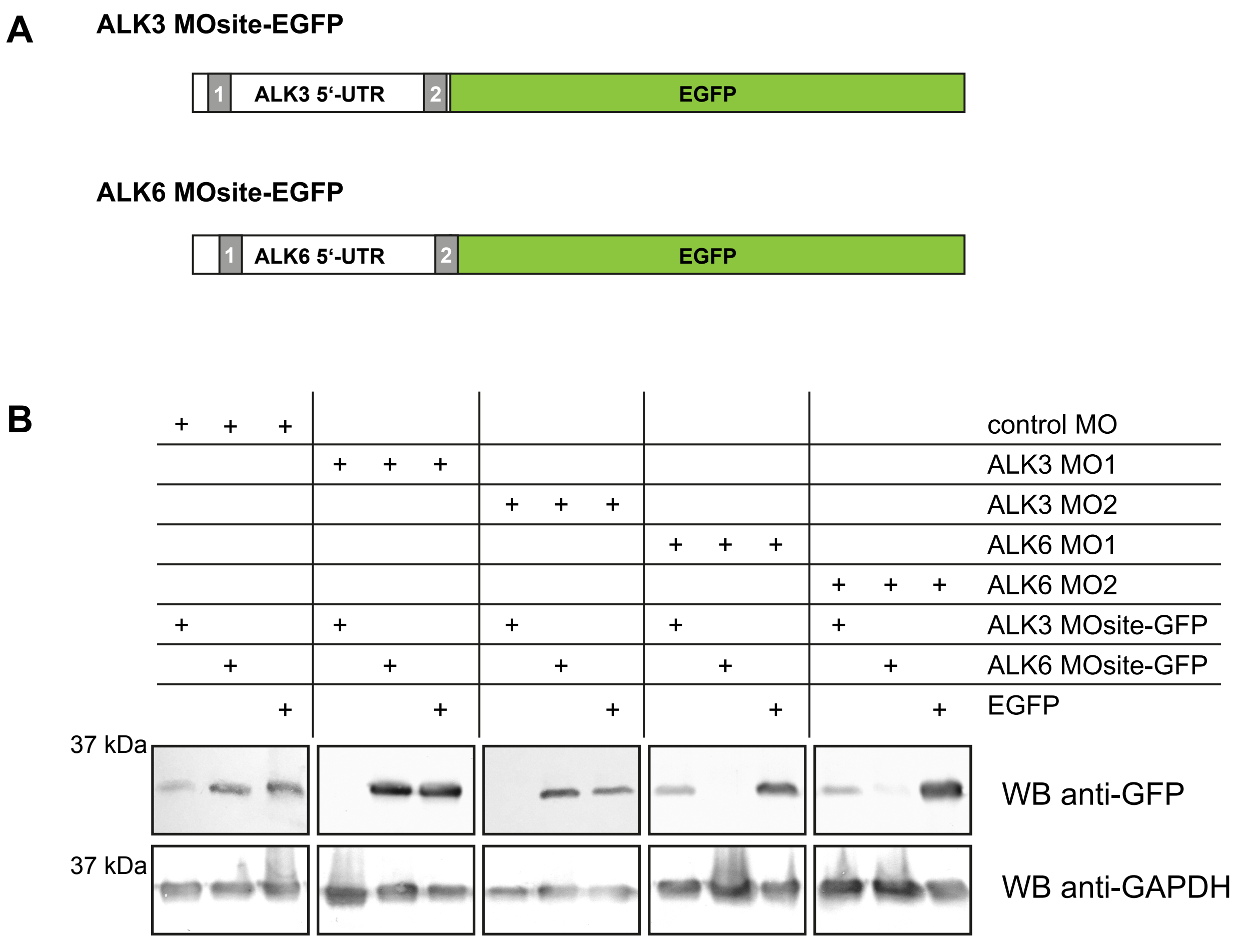

Additional Figure 4. ALK3 MO and ALK6 MO specificity. (A) The 5‘ UTR including the MO binding site of *alk3* and *alk6* respectively was fused to the coding sequence of *egfp* and cloned into the pCS2 vector. Grey boxes indicate MO binding sites. (B) The synthetic fusion constructs or pCS2-EGFP were co-injected with a control MO, ALK3 MO 1, ALK3 MO 2, ALK6 MO 1 and ALK6 MO 2 into both blastomeres of two-cell-stage embryos as indicated. Embryos were grown to NF stage 11, lysed and GFP expression detected on Western Blots. Endogenous GAPDH served as loading control.

One set of blots from a total of five experiments is shown. Control MO did not affect expression of EGFP from either construct; it should be noted that expression of ALK3 MOsite-GFP was weaker in all samples, which could be due to less efficient translation or shorter life-time of this particular RNA. ALK3 MO specifically and efficiently blocked translation of the ALK3 MOsite-EGFP fusion construct, but not that of ALK6 MOsite-EGFP and vice versa demonstrating the specificity of all four antisense MOs.
