# Supplementary material for: Differential requirement of bone morphogenetic protein receptors Ia (ALK3) and Ib (ALK6) in early embryonic patterning and neural crest development
Source: BMC Dev Biol. 2016 Jan 19;16:1. doi: 10.1186/s12861-016-0101-5 (PMC4717534; doi:10.1186/s12861-016-0101-5)
Supplement: Additional file 5: Figure S5. — ALK6 morphant embryos showed a lower survival rate. (PDF 116 kb) [file 12861_2016_101_MOESM5_ESM.pdf]

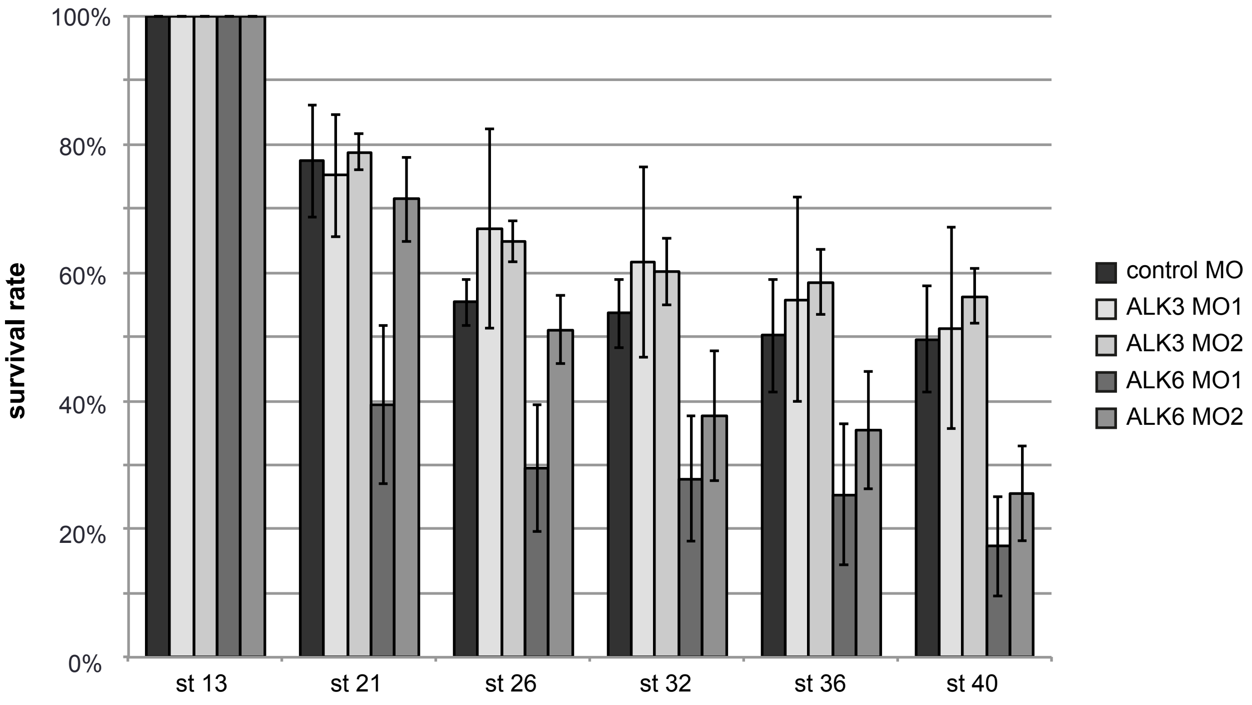

Additional Figure 5. ALK6 morphant embryos showed a lower survival rate. Embryos were injected in both blastomeres at the two-cell stage with the indicated MOs. During gastrulation, comparable lethality was observed in all groups (not shown). After gastrulation, fewer ALK6 MO injected embryos survived until tadpole stages and an additional 10% of these embryos died between stage 36 and 40 indicating a putatively vital role of ALK6 in this phase of development. Survival of ALK3 morphant embryos after gastrulation was very similar to that of embryos injected with control MO.

The graph shows the average proportion (± SEM) of surviving embryos from three independent injections at the indicated stages.
