# Supplementary material for: Differential requirement of bone morphogenetic protein receptors Ia (ALK3) and Ib (ALK6) in early embryonic patterning and neural crest development
Source: BMC Dev Biol. 2016 Jan 19;16:1. doi: 10.1186/s12861-016-0101-5 (PMC4717534; doi:10.1186/s12861-016-0101-5)
Supplement: Additional file 6: Figure S6. — Knock-down of ALK3 in the dorsal ectoderm results in an expansion of neural tissue. (PDF 159 kb) [file 12861_2016_101_MOESM6_ESM.pdf]

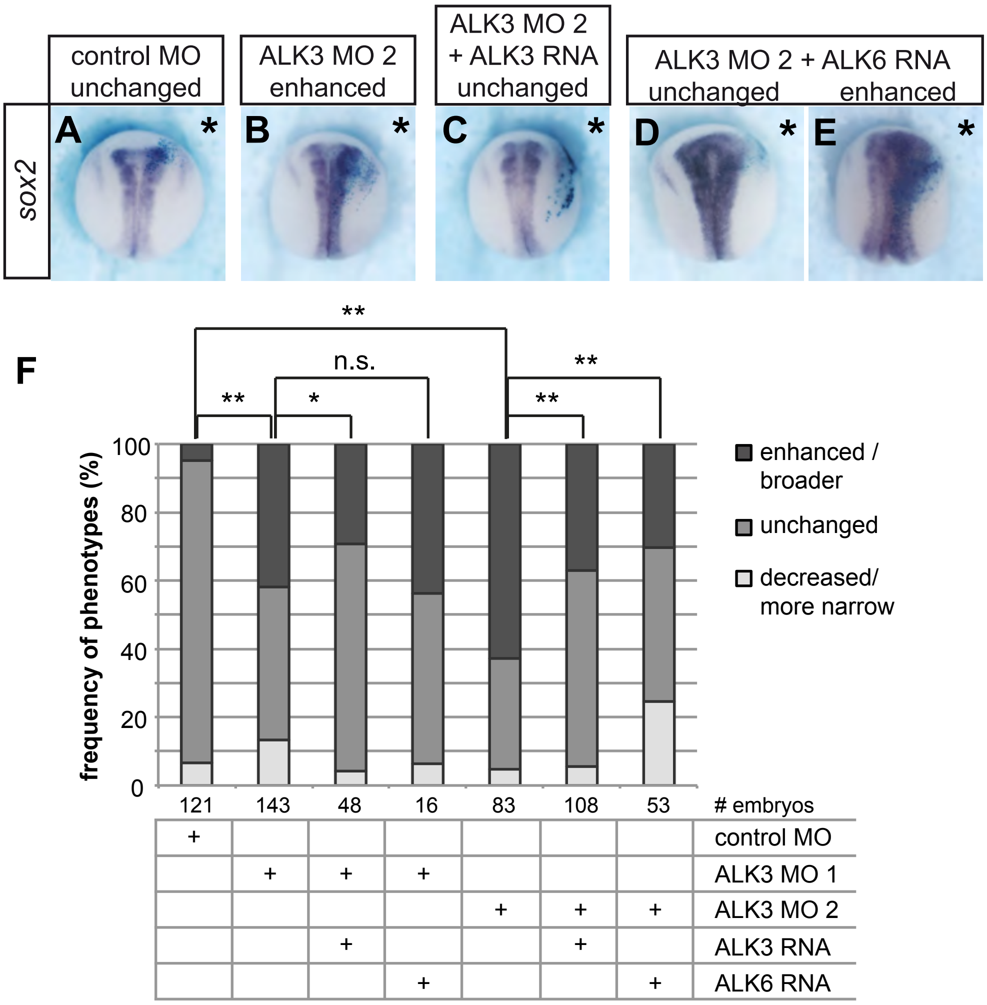

Additional Figure 6. Knock-down of ALK3 in the dorsal ectoderm results in an expansion of neural tissue. Eight-cell stage embryos were injected into one animal-dorsal blastomere to target the dorsal ectoderm. Embryos were injected with 0.8 pg of ALK3 MO 1 or ALK3 MO 2 and, for rescue experiments, co-injected with 50 pg of *alk3* RNA or *alk6* RNA. (A-E) Representative examples of embryos injected as indicated; the injected side was identified by LacZ and is labelled with an asterisk. (F) The graph summarizes the results from four independent experiments.
