# Supplementary material for: Differential requirement of bone morphogenetic protein receptors Ia (ALK3) and Ib (ALK6) in early embryonic patterning and neural crest development
Source: BMC Dev Biol. 2016 Jan 19;16:1. doi: 10.1186/s12861-016-0101-5 (PMC4717534; doi:10.1186/s12861-016-0101-5)
Supplement: Additional file 7: Figure S7. — Phenotypes of ALK3 and ALK6 morphant embryos are rescued with alk3 or alk6 RNA. (PDF 361 kb) [file 12861_2016_101_MOESM7_ESM.pdf]

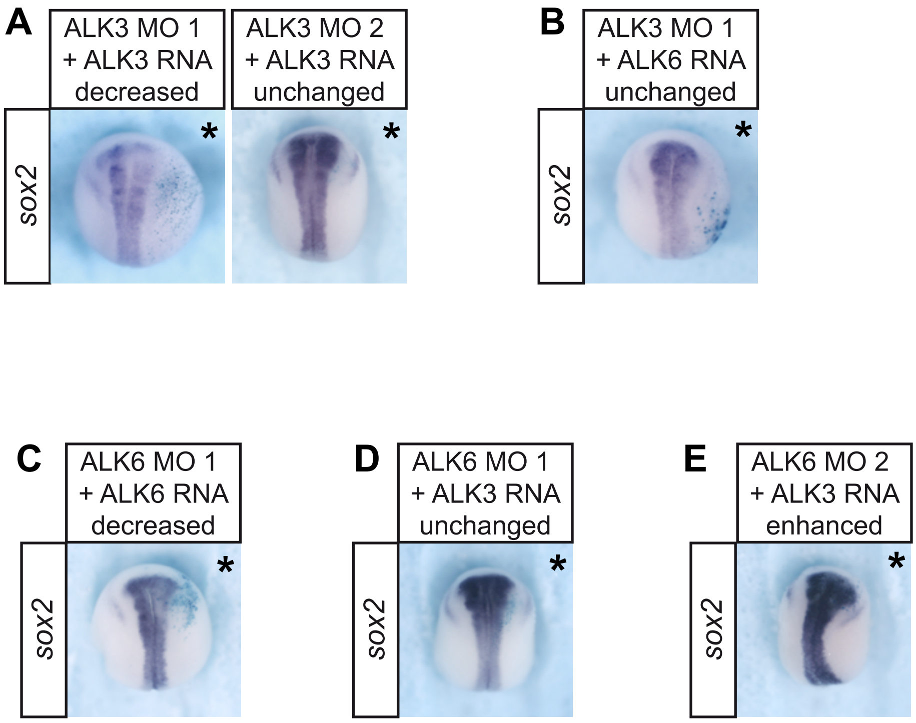

Additional Figure 7. Phenotypes of ALK3 and ALK6 morphant embryos are rescued with *alk3* or *alk6* RNA. Four-cell stage embryos were injected into one dorsal blastomere. (A-E) Examples of embryos injected as indicated each representing one observed phenotype; the injected side was identified by LacZ and is labelled with an asterisk. The summary of phenotype frequencies is provided in Figure 4.
