# Supplementary material for: Differential requirement of bone morphogenetic protein receptors Ia (ALK3) and Ib (ALK6) in early embryonic patterning and neural crest development
Source: BMC Dev Biol. 2016 Jan 19;16:1. doi: 10.1186/s12861-016-0101-5 (PMC4717534; doi:10.1186/s12861-016-0101-5)
Supplement: Additional file 8: Table S1. — Primer sequences for RT-PCR and cloning. (PDF 51 kb) [file 12861_2016_101_MOESM8_ESM.pdf]

Supplementary Table 1
Primer sequences for RT-PCR and cloning

**Primer name**	**Sequence**	**Reference**
alk4-fwd	agctgattgcaacaatgcctaa	
alk4-fwd	tttagattgcctgaaagcgaga	
alk2-fwd	ttggtcttgtcctatgggaggt	
alk2-rev	tttgatgcgtaaggctgtgagt	
vent1-fwd	aaactggaacaggcattcaaca	
vent1-rev	ttctaacatggtggtggagtgg	
bmpr2-fwd	cctgaccctttaagcagcaca	
bmpr2-rev	gctagccaagcagctgatattggcttct	
alk3-fwd	cagcttgtggattgtgtcacct	
alk3-rev	ggcatttcatgcagacaacttc	
alk6-fwd	tgcttatgcgacatgaaaac	
alk6-rev	ggtatgtccacttcattcgtg	
acvr2a-fwd	tcagttccaccttctcacgaaa	
acvr2a-rev	taaggccgtatctggaacacct	
admp-fwd	gatcaccatgaaagcaaacagc	
admp-rev	atagactgcacagtggcatggt	
bmp4-fwd	tgacacgggcaagaagaaagta	
bmp4-rev	atgctgatatcgtgcagctcat	
chordin-fwd	cctccaatccaagactccagcag	Sasai et al. (1994)
chordin-rev	ggaggaggaggagctttgggacaag	Sasai et al. (1994)
gsc-fwd	cgtgttgtggagcagttcaag	
gsc-rev	aagggagcatctggtgagg	
sizzled-fwd	ctgatacggaacagacccacag	
sizzled-rev	tcttcagcatggtaatgggatg	
alk3-fwd2	ttgcctttcacattctcttcaca	
alk3-rev2	tgaagctgccaatttctttcttc	
alk6-fwd2	aacacttgcaaagatggtggagt	
alk6-rev2	tagtacacacaggctcccactga	
alk3-cloning-fwd	gaattcatgagaaaacgacttttcattgcat	
alk3-cloning-rev	gctagcaatcttgacgtcctgggact	
alk6-cloning-fwd	gaattcatgggttggttggaagaaaga	
alk6-cloning-rev	gctagcgagcttgatatcttgagactctga	
